# Therapeutical Administration of Peptide Pep19-2.5 and Ibuprofen Reduces Inflammation and Prevents Lethal Sepsis

**DOI:** 10.1371/journal.pone.0133291

**Published:** 2015-07-21

**Authors:** Lena Heinbockel, Sebastian Marwitz, Sergio Barcena Varela, Raquel Ferrer-Espada, Norbert Reiling, Torsten Goldmann, Thomas Gutsmann, Walter Mier, Tobias Schürholz, Daniel Drömann, Klaus Brandenburg, Guillermo Martinez de Tejada

**Affiliations:** 1 Clinical & Experimental Pathology, Research Center Borstel, Leibniz-Center for Medicine and Bioscience, Borstel, Germany; 2 Department of Microbiology and Parasitology, University of Navarra, Pamplona, Spain; 3 Microbial Interface Biology, Research Center Borstel, Leibniz-Center for Medicine and Bioscience, Borstel, Germany; 4 Biophysics, Research Center Borstel, Leibniz-Center for Medicine and Bioscience, Borstel, Germany; 5 Department of Nuclear Medicine, Heidelberg University Hospital, Heidelberg, Germany; 6 Department of Intensive Care, University Hospital Aachen, Aachen, Germany; 7 Medical Clinic III, University of Schleswig-Holstein, Lübeck, Germany; 8 Airway Research Center North (ARCN), Member of the German Center for Lung Research (DZL), Großhansdorf, Germany; University of Leuven, Rega Institute, BELGIUM

## Abstract

Sepsis is still a major cause of death and many efforts have been made to improve the physical condition of sepsis patients and to reduce the high mortality rate associated with this disease. While achievements were implemented in the intensive care treatment, all attempts within the field of novel therapeutics have failed. As a consequence new medications and improved patient stratification as well as a thoughtful management of the support therapies are urgently needed. In this study, we investigated the simultaneous administration of ibuprofen as a commonly used nonsteroidal anti-inflammatory drug (NSAID) and Pep19-2.5 (Aspidasept), a newly developed antimicrobial peptide. Here, we show a synergistic therapeutic effect of combined Pep19-2.5-ibuprofen treatment in an endotoxemia mouse model of sepsis. *In vivo* protection correlates with a reduction in plasma levels of both tumor necrosis factor α and prostaglandin E, as a likely consequence of Pep19-2.5 and ibuprofen-dependent blockade of TLR4 and COX pro-inflammatory cascades, respectively. This finding is further characterised and confirmed in a transcriptome analysis of LPS-stimulated human monocytes. The transcriptome analyses showed that Pep19-2.5 and ibuprofen exerted a synergistic global effect both on the number of regulated genes as well as on associated gene ontology and pathway expression. Overall, ibuprofen potentiated the anti-inflammatory activity of Pep19-2.5 both *in vivo* and *in vitro*, suggesting that NSAIDs could be useful to supplement future anti-sepsis therapies.

## Introduction

Sepsis is one of the leading causes of death in hospitalized patients worldwide. Current estimations calculate 800,000 cases of severe sepsis annually only in United States, potentially rising to 1,600,000 cases by 2050 [1]. There are indications, that non-steroidal anti-inflammatory drugs (NSAIDs) modify the course of the disease [2], but it remains controversial whether their administration to sepsis patients is beneficial. NSAIDs are one of the most widely used and best selling medicines, due to their analgesic and anti-inflammatory effect. These drugs inhibit the activity of the prostaglandin-synthesizing enzyme cyclooxygenase-2 (COX-2) and most of them also the activity of the cyclooxygenase-1 (COX-1).

One of those frequently used non-selective COX inhibitors is ibuprofen. Besides its pain-relieving and anti-inflammatory properties, ibuprofen also possesses anti-pyretic activity. However, treatment of septic patients with ibuprofen has not been reported to reduce significantly their mortality [3]. This is in contrast to results from various animal models where a survival benefit was shown in endotoxemic mice or rabbits treated with ibuprofen [4, 5].

Generally, sepsis is a systemic pathology caused by the invasion of microorganisms or their toxins into the bloodstream. Recognition of microbial components like lipopolysaccharide (LPS; endotoxin) by specific immune cell receptors induces secretion of pro-inflammatory cytokines, such as Tumor Necrosis Factor α (TNFα). Whereas this response is in general instrumental to control infection, excessive activation of immune cells can lead to septic shock, which is frequently lethal [6].

The primary site of infections leading to sepsis can be manifold with lung, gut and urinary tract infections having the highest incidence [7]. Just as diverse as the causes are the clinical progressions of the disease. Consequently, the simulation of sepsis in the animal models is challenging and the validity of the different available models is always questioned [8, 9]. However, regardless of the different models and species, the positive effect of the COX-inhibition in hemodynamic parameters as well as in survival was consistently reported. A comprehensive discussion about the various animal models of sepsis and potential reasons for their discrepancy with regard to human studies was put forward by D. M. Aronoff [10].

Two different mouse models of sepsis, namely the endotoxemia and the cecal ligation and puncture model, were used to demonstrate the potent endotoxin-neutralising effects of our secondly investigated drug, the peptide Pep19-2.5 [11, 12]. This synthetic compound belongs to a class of short cationic peptides called host-defense peptides or antimicrobial peptides (AMPs). AMPs are characterized by their ability to bind to conserved anionic components of the microbial envelope, such as LPS in Gram-negative bacteria, and to disrupt or perturb microbial membranes [13–15].

AMPs are attractive candidates for the development of new drugs, due to the low likelihood of resistant mutant emergence, their fast mechanism of killing and their controlled biodegradability [16]. However, to efficiently kill the invading microorganisms during sepsis does not necessarily imply to stop the progression of the disease [17]. Lysed bacteria can release vast amounts of toxic components, such as endotoxins and lipoproteins, which belong to the most potent immune stimulators known.

In our current study, we stimulated mice with endotoxin and demonstrated that the synergistic effect of ibuprofen and Pep19-2.5 reduces the pro-inflammatory cytokine serum levels and increases the overall survival of the animals. To complement this relevant *in vivo* data we performed a transcriptome analysis of LPS-stimulated human monocytes. Results from gene expression experiments confirmed the findings from murine *in vivo* results and showed a clear benefit of the combined application of both drugs on the host response to LPS.

## Material and Methods

### LPS purification

LPS used throughout this project was isolated from *Salmonella enterica* Serovar Minnesota R60 [18] by applying the traditional phenol:water method [19] and purified according to [20, 21]. To promote LPS solubilisation, 3 μl of triethylamine (MERCK, Madrid, Spain) were added to each ml of LPS suspension [22]. To induce a uniform aggregation state and to promote interassay reproducibility LPS was subjected to three consecutive cycles of heating and cooling (56°C for 15 min.; 5 min. on ice) prior to each experiment.

### Peptide synthesis

For preliminary tests the peptide Pep19-2.5 (Aspidasept: Amino acid sequence: GCKKYRRFRWKFKGKFWFWG) was synthesized with an amidated C terminus by the solid-phase peptide synthesis technique in an automatic peptide synthesizer (model 433A; Applied Biosystems) on Fmoc-Rink amide resin, according to the 0.1-mmol FastMoc synthesis protocol of the manufacturer, including the removal of the N-terminal Fmoc group. For the final experiments Pep19-2.5 was synthesized commercially (BACHEM, Bubendorf, Switzerland, Lot No. 1053821). The purity of all applied peptides was above 90%.

### Animal model of endotoxic shock

All the animal experiments were approved by the Animal Research Committee of University of Navarra (Protocols 069–09 y E6-11) and were carried out in female 7 week-old Balb/c mice weighting approximately 20 g (Harlan Interfauna Iberica S.A., Barcelona, Spain). Mice were randomly caged in groups of six with access to food and water *ad libitum* and light-dark cycles of 12 h. After one week of acclimatization, animals were intraperitoneally inoculated with 400 μg of LPS of *S*. *enterica* Serovar Minnesota R60 dissolved in 200 μl of sterile saline solution (0.9% w/v of NaCl),. After 1 h of LPS challenge, either ibuprofen (4 μg/mouse) or Pep19-2.5 (400 μg/mouse) or the combination of both compounds dissolved in sterile saline was intraperitoneally administered. In independent assays, the same treatments were given at different times with respect to endotoxin inoculation (30 min. before LPS, or immediately after LPS challenge). During the experiments, the condition of the animals was monitored daily every 6 h and animals displaying persistent motor ataxia and hunched posture were euthanized. For this purpose, mice were sacrificed by cervical dislocation by a technician with a demonstrated high degree of technical proficiency.

#### Mouse serum cytokine measurement

Levels of cytokines were determined in animal serum 2h after LPS administration. Blood samples were centrifuged (10.000 x *g*, 5 min.), coagulated blood was discarded and serum was transferred to clean cryotubes for immediate ELISA analysis or stored for further experiments at -80°C. For TNFα quantification, an ELISA commercial kit was used (Mouse TNFα, Quantikine ELISA Kit, R&D Systems, Madrid) following manufacturer’s instructions. For prostaglandin E2 (PGE2) analysis, a final concentration of 10 μM indomethacin (Sigma-Aldrich, Haverhill, United Kingdom) was added to the serum immediately after extraction to block COX activity and prevent ex-vivo formation of prostaglandin. PGE metabolites were quantified using a commercial system (Prostaglandin E Metabolite ELISA Kit, Cayman Chemical Company, Michigan, USA). This technique required special sample purification, derivatization and acidification prior to performing the assay, as described in the manufacturer’s protocol. Those steps were necessary for converting all PGE metabolites into stable compounds that were measurable.

#### Statistical analysis of the mouse experiments

Statistical analysis was performed using Graph Pad Prism 5 software (GraphPad Software, La Jolla, CA). Statistical differences between groups (data for 6 or 8 mice per treatment or 3 or 5 independent *ex vivo* experiments) were analysed by Mann-Whitney U Test or One-Way Analysis of Variance (ANOVA) followed by Tukey’s multiple comparison test (*, *P <* 0.05; **, *P <* 0.01). All data are shown as the mean ± standard error of the mean (SEM).

### Stimulation of human monocytes

Monocytes were used for LPS stimulation experiments to reflect a sensitive human blood cell type involved in the inflammatory onset of sepsis. Briefly, cells were isolated from heparinized blood of 3 healthy donors by the Hypaque-Ficoll density gradient method [23]. Monocytes were subsequently purified from PBMC by counterflow elutriation (purity consistently greater than 95%) [24]. The cell number was adjusted to 5 x 10^5^ cells/ml RPMI 1640 containing 2 mM L-glutamine, 100 U/ml penicillin and 100 μg/ml streptomycin. For stimulation, 200 μl of the monocyte cell suspension was transferred into 96-well culture plates. The stimuli were serially diluted in RPMI 1640 and added to the cultures at 20 μl per well. Concentrations of 0.1 ng/ml LPS R60 from *Salmonella enterica* Minnesota rough mutants, 10 ng/ml Pep19-2.5 (Bachem, Lot. 1053821) and 1 μg/ml ibuprofen (Fluka 11892, Lot. BCBI499V) were applied in 0.9% NaCl. The cultures were incubated for 4 h at 37°C under 5% CO_2_. Cell-free supernatants were collected after centrifugation of the culture plates for 10 min. at 400 x g and stored at –20°C until determination of the cytokine content. Immunological determination was performed in the cell supernatant in duplicate by using the OptEIA ELISA (BD, Heidelberg, Germany) for TNFα and for the determination of PGE2 the PGE2 ELISA Kit (Thermo Scientific EHPGE2, Rockford, USA). Monocytes for the extraction of RNA were stimulated under the same conditions as described above.

#### Statistical analysis of the stimulation of human monocytes

Statistical analysis was performed using Graph Pad Prism 5 software (GraphPad Software, La Jolla, CA). Data of 3 different donors were analysed by using one-way ANOVA with Bonferroni correction as post-hoc test (*p < 0.5, **p < 0.01, ***p < 0.01). All data are shown as mean ± SEM.

#### RNA isolation

Total RNA from stimulated human monocytes was extracted using the RNeasy Mini Kit (Qiagen, Hilden, Germany) according to manufacturer´s instructions and stored at -80°C until analysis.

#### Transcriptome analysis

For microarray analysis, a total of 21 samples from 3 biological replicates were used for evaluation. Quality control of total RNA samples was conducted using the Agilent Bioanalyzer with the RNA 6000 Nano Kit (Agilent, Waldbronn, Germany). For cDNA synthesis, amplification and labelling with Cy3 during reverse transcription by T7 RNA polymerase, the Low Input Quick Amp labelling kit (Agilent, Waldbronn, Germany) was used according to manufacturer´s instructions. Cy3-labelled cRNA was purified using the RNeasy Mini Kit (Qiagen, Hilden, Germany) and the specific activity was calculated using a NanoDrop 2000 spectrophotometer (Thermo Fisher Scientific, Waltham, MA, USA). For each sample 1650 ng of labelled cRNA was hybridized to Agilent Human Whole Genome Gene Expression 4x44k V2 arrays and scanned with an Agilent SureScan microarray scanner at a resolution of 5μm. Raw data was extracted applying the 1-color gene expression protocol from within the Agilent Feature Extraction Software v11. GeneSpring software version 13 was used for statistical analyses (One-Way ANOVA with Tukey HSD post hoc test and Benjamini-Hochberg multiple testing correction, cut-off p≤0.05) on probe-level experiment. Before statistical evaluation, intrinsic controls were used to remove compromised and non-detected probes from data set. For displaying expression levels of selected genes (based on statistical evaluation within GeneSpring), the raw data were quantile-normalized according to Bolstad et al. [25] using the DirectArray software (Oaklabs, Hennigsdorf, Germany). Gene ontology terms for lists of genes were identified by the Gorilla web application [26] using a p-value cut-off of 10^−7^ and the default options. All entities present on the Agilent 4x44K V2 arrays served as background list for analysis of GO term enrichment. Enrichment for pathways was conducted within GeneSpring software from Wikipathways Analysis, Reactome and Biocyc databases (cut-off of ≤0.05) from significantly up-regulated genes (Fold Change≥2,.p≤0.05) against medium control.

The complete dataset has been deposited at Gene Expression Omnibus under ID GSE65855 and will be released upon publication.

## Results

### Anti-endotoxic activity of ibuprofen and Pep19-2.5 in mice

**Fig 1:**
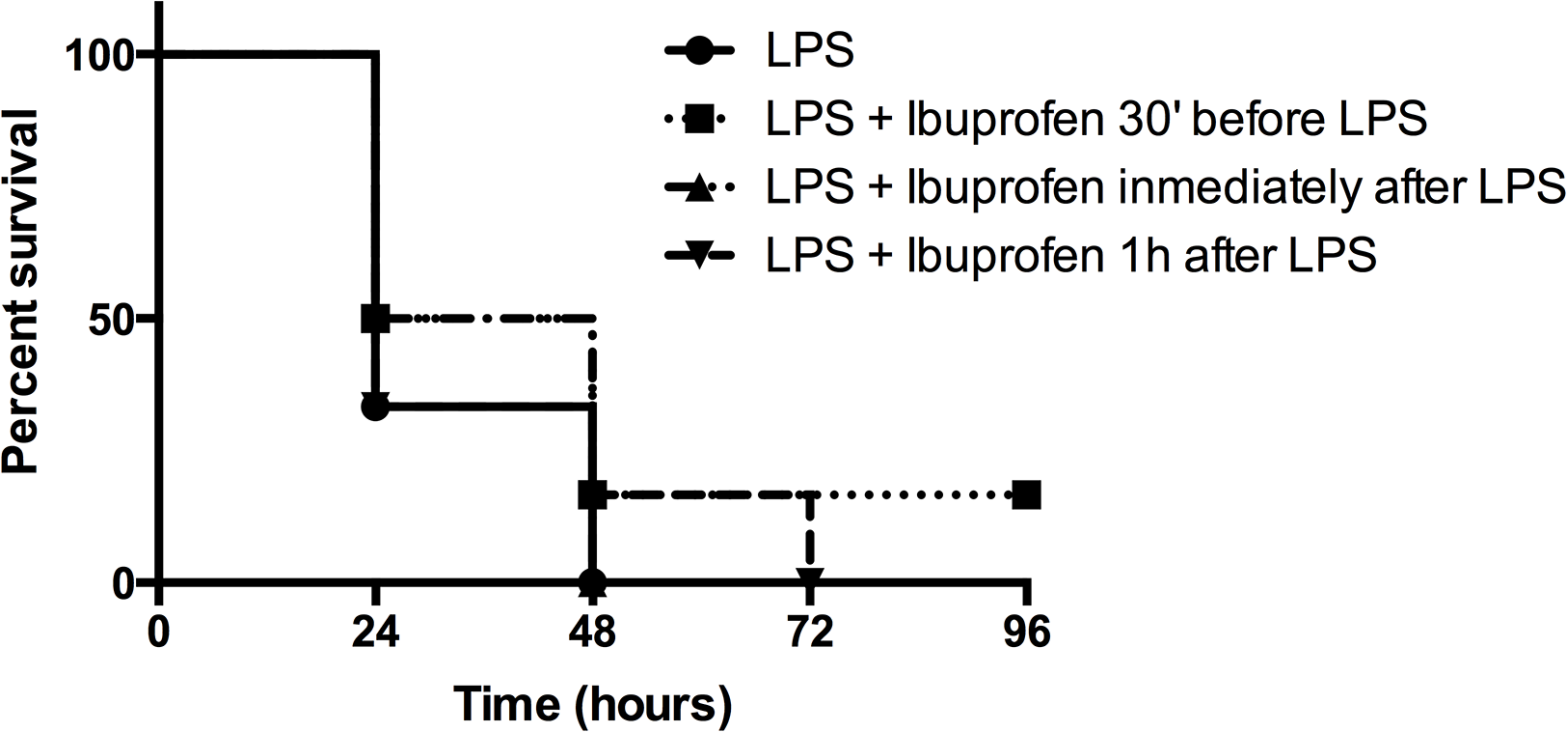
Administration of Ibuprofen to mice before or after LPS challenge has no protective effect. Kinetics of mortality of groups of mice (n = 6) intraperitoneally challenged with LPS from *S*. *enterica* Serovar Minnesota R60 (400 μg/mouse) and treated with (40 μg/mouse) of ibuprofen at different time points with respect to LPS inoculation (30 minutes before or immediately afterwards or 1 h subsequent to challenge). A control group (labeled as “LPS”) received only 400 μg/mouse of LPS. The Kaplan Meier test was used for statistical analysis.

In preliminary experiments, we found that intraperitoneal (i.p.) inoculation of 400 μg of *S*. *enterica* Serovar Minnesota R60 LPS caused a mortality of 90% in mice at 48 h post-inoculation. To study if ibuprofen could protect animals against endotoxic shock we inoculated i.p. the compound (40 μg/mouse) at different time-points with respect to LPS challenge. As shown in Fig 1, neither prophylactically nor therapeutically administered ibuprofen improved the survival of mice.

**Fig 2:**
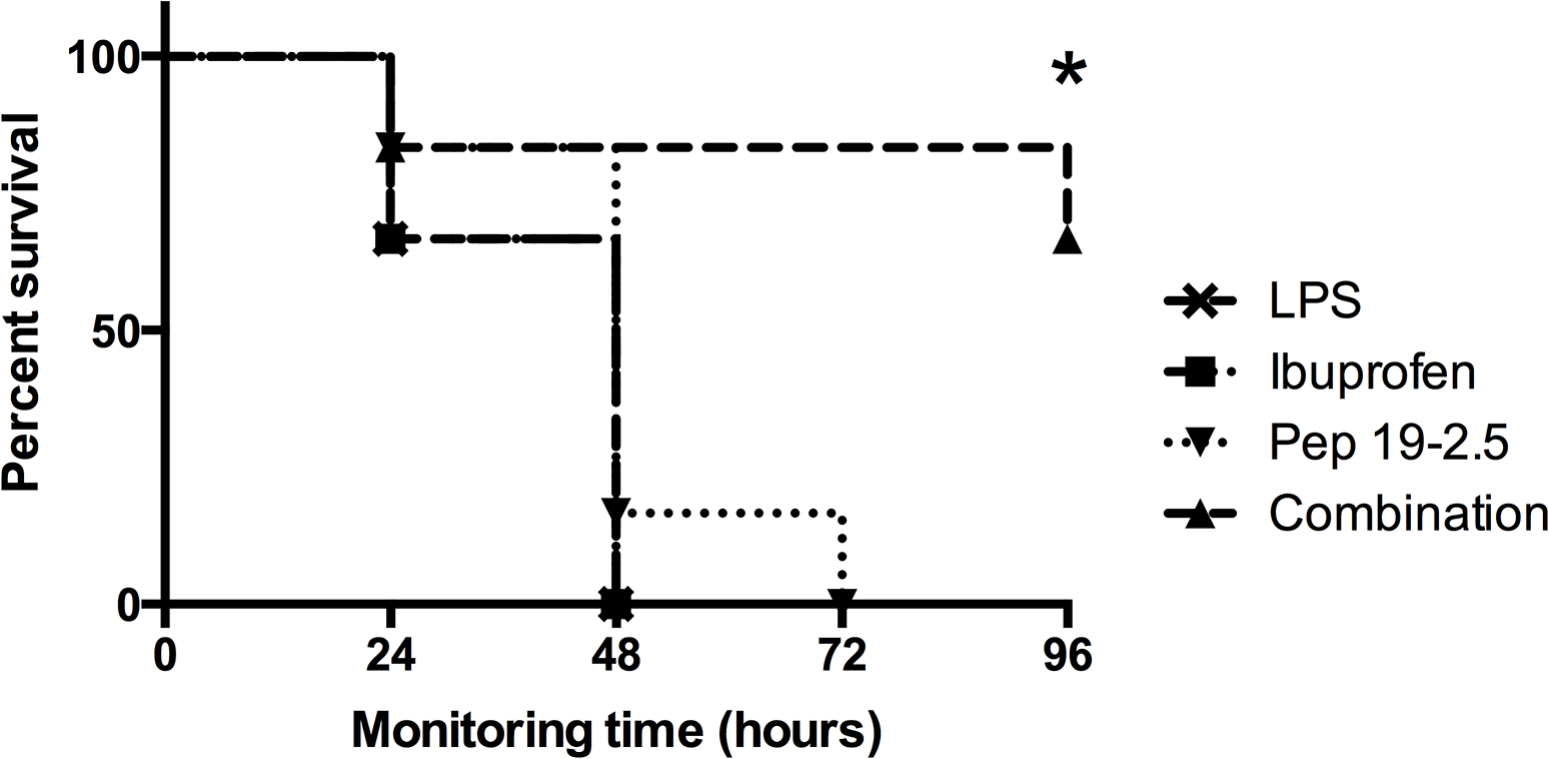
Pep19-2.5 acts in synergy with ibuprofen and protects mice against lethal endotoxemia. Kinetics of mortality of groups of mice (n = 6) intraperitoneally inoculated with LPS from *S*. *enterica* Serovar Minnesota R60 (400 μg/mouse) and treated 1 h later with either ibuprofen (40 μg/mouse), or Pep19-2.5 (400 μg/mouse) or a combination of both treatments. A control group (labelled as “LPS”) received only 400 μg/mouse of LPS. The Kaplan Meier test was used for statistical analysis.

Pep19-2.5 was shown to protect mice against endotoxic shock, although protection progressively diminished if treatment with the peptide was delayed with respect to LPS challenge [11]. Because ibuprofen and Pep19-2.5 are expected to block different pro-inflammatory pathways (COX and TLR4 dependent cascades, respectively), we hypothesized that these compounds could act in synergy *in vivo* to protect against septic shock. To investigate such potential synergistic activity we examined whether ibuprofen could enhance the poor Pep19-2.5 anti-endotoxic performance previously observed at late time points. As shown in Fig 2, administration of a single dose of Pep19-2.5 combined with ibuprofen conferred long-term protection against mortality due to septic shock. Notably, none of the treatments was protective when given alone.

### Pep19-2.5 acts mainly on TNF-α rather than PGE2 in the murine endotoxemia model

To study if there is a correlation between animal survival and inhibition of pro-inflammatory cytokines the experiment shown in Fig 2 was repeated and serum levels of TNFα and PGE2 were determined by ELISA 2 h after LPS challenge. Blood was obtained from the retro orbital plexus of anesthetized animals and then exsanguinated mice were sacrificed. Due to its very short half-life *in vivo*, PGE2 levels were measured indirectly by quantifying its metabolic products (“PGE metabolites”; see Material and Methods).

**Fig 3:**
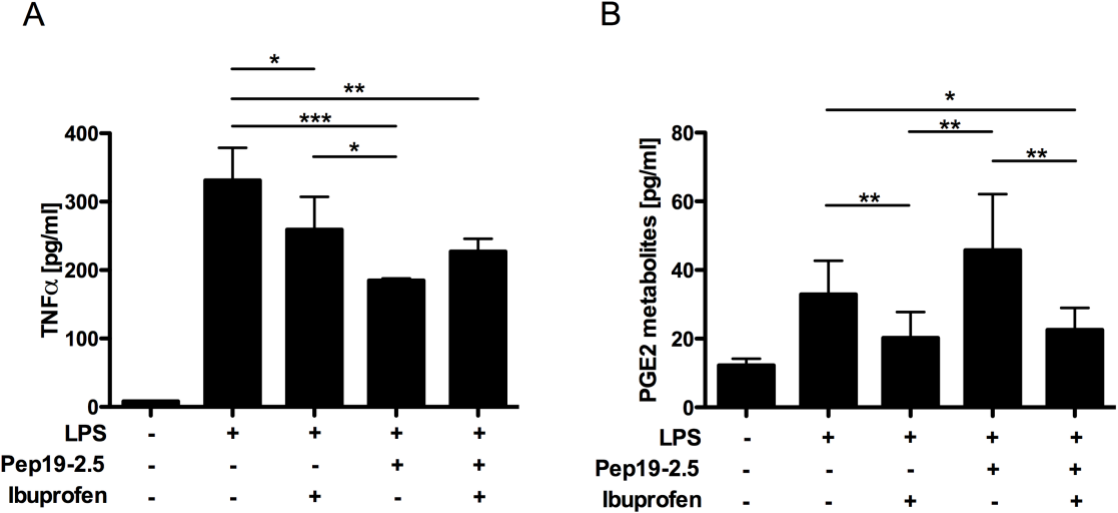
Animals receiving the combined ibuprofen-Pep19-2.5 treatment have low serum levels of both TNFα and PGE 2h after LPS administration. Plasma TNFα (pg/ml) (A) and PGE Metabolites (pg/ml) (B) levels in groups of mice (at least n = 6) intraperitoneally inoculated with LPS from *S*. *enterica* Serovar Minnesota R60 (400 μg/mouse). Groups received the same treatments as detailed in Fig 2. Negative control group (n = 3) received neither LPS nor treatment. Statistical differences were analysed using ANOVA followed by DMS (parametrical, A) and Thamhane (no parametrical, B as there was heterogeneity of variances) post hoc tests (*, *P <* 0.05; **, *P <* 0.01; *** p<0.001). Very significant statistical differences (p<0.01 for PGE2 or p<0.001 for TNFα) were found between the negative control group (labelled with the symbol #) and the rest of the groups.

As shown in Fig 3A, all the experimental treatments reduced TNFα levels in comparison to those measured in LPS challenged mice left untreated. This reduction was more pronounced in the case of animals receiving either the combined treatment (p<0.01) or Pep19-2.5 (p = 0.002). Mice treated with ibuprofen had significantly higher levels of TNFα (p = 0.016) than the Pep19-2.5 treated group. However, addition of the peptide to ibuprofen (i.e. the combined treatment) did not enhance the ability of the NSAID to decrease TNF-α any further. Regarding the activity of the COX-dependent pathway, as shown in Fig 3B, LPS-induced PGE metabolites levels decreased upon treatment with ibuprofen (LPS *vs*. LPS+ ibuprofen (p = 0.020)); LPS *vs*. combined treatment (p = 0.021)). In contrast, presence of Pep19-2.5 alone or in combination with ibuprofen appeared to have little influence on the levels of PGE metabolites.

### Pep19-2.5 acts mainly on TNF-α rather than PGE2 response of human monocytes to LPS

**Fig 4:**
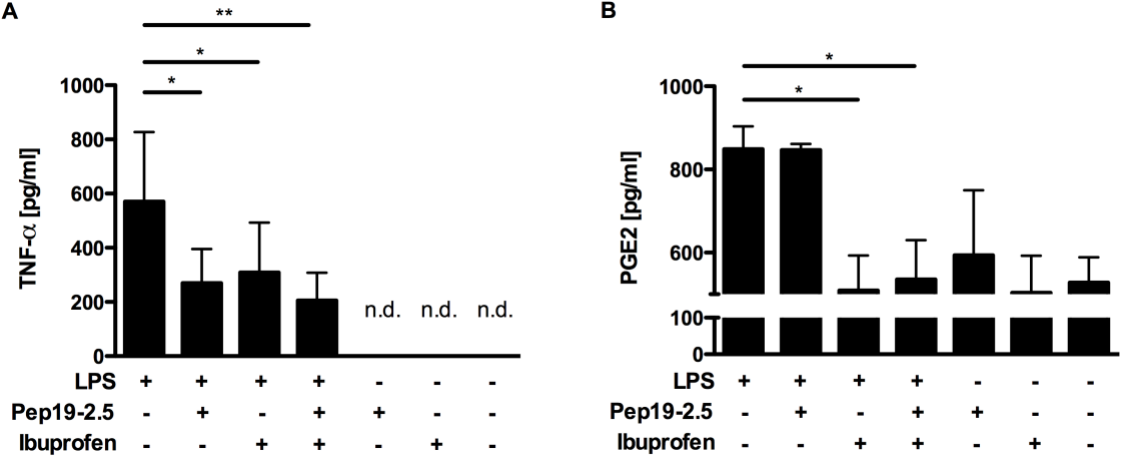
The secretion levels of the inflammatory markers TNFα and PGE2 after LPS stimulation. Expression levels in cell supernatants of TNFα(pg/ml) (A) and PGE2 (pg/ml) (B) of human monocytes after 4 h and 24 h of stimulation, respectively. Not detectable n.d. One-way ANOVA with Bonferroni correction as post-hoc test (*p < 0.5, **p < 0.01, ***p < 0.01) was used to statistically analyse cells from 3 different donors. All data are shown as mean ± SEM.

Human monocytes were used for the stimulation and closer investigation of the LPS-induced effects on human cells. For a comparison with the mouse data we first analysed the levels of TNFα and PGE2 in the supernatants of the stimulated cells (Fig 4). The results revealed a similar picture for the *in vitro* as for the *in vivo* experiments. Both, the addition of Pep19-2.5 as well as the addition of ibuprofen led to a decreased TNFα response compared to LPS without additives (Fig 4A). This effect was slightly amplified in the combinational treatment. On the contrary, no effect was observed for the PGE2 level after peptide treatment, while a strong decrease in PGE2 was observed for the ibuprofen addition, as expected (Fig 4B).

### Treatment with Pep19-2.5 and ibuprofen downregulates the global gene expression response to LPS of monocytes

**Fig 5:**
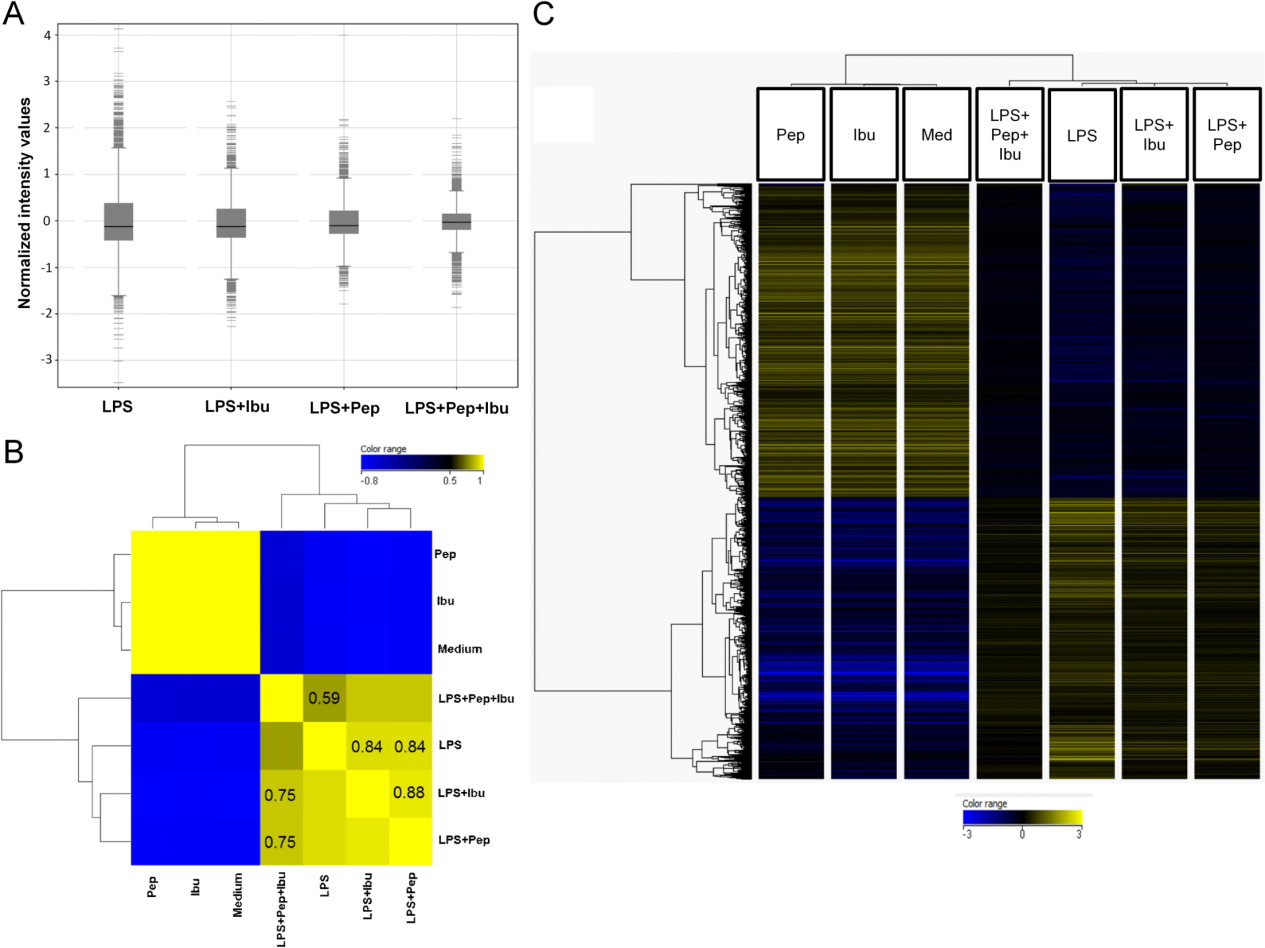
The combination of ibuprofen and Pep19.2–5 alleviates the transcriptional LPS-response of human monocytes. Gene expression analysis of human monocytes stimulated with LPS, ibuprofen, Pep and combinations of both after 4 h of stimulation. Samples from 3 biological replicates of different donors were analysed by 1W-ANOVA with Tukey HSD post hoc testing for detection of differential expressed genes between parameters with a Benjamini-Hochberg multiple testing correction (cut-off ≤0.05). A: Variance in gene expression as indicated by the mean value of gene expression as well as its range with regard to LPS, LPS+Ibu, LPS+Pep and LPS+Ibu+Pep stimulation. The variance in gene expression upon LPS-stimulation is reduced by treatment with anti-inflammatory drugs ibuprofen, Pep or both. B: Pearson correlation analysis of significantly regulated genes from 1W-ANOVA analysis with subsequent hierarchical clustering (squared Euclidian distance; Ward`s linkage rule) on experimental conditions ordered the samples according to their similarity. Correlation coefficients are colour coded and exact numbers provided inside the boxes. C: Hierarchical clustering of significantly regulated genes from those genes that were found to be significantly regulated by LPS were subjected to Hierarchical Clustering (Pearson Centered distance matrix, Wards Linkage Rule) and relative gene expression is displayed for all experimental parameters as a heatmap.

Transcriptomes of challenged human monocytes were analysed by One-Way ANOVA with Tukey post hoc testing (cut-off p≤0.05) to identify significant differentially expressed genes between experimental groups (see S1 Table). Since the animal survival experiments suggested a possible synergistic effect of Pep19-2.5 and ibuprofen if applied in combination, the microarray data was further evaluated for this purpose. The variance in global gene expression with regards to their range in intensity is plotted in Fig 5A. In general, LPS-stimulation led to more regulated genes which were also found to be more strongly expressed than co-stimulation with ibuprofen or Pep19-2.5 alone. The combination of both compounds with LPS resulted in a decrease of gene expression intensity as well as in the number of highly expressed genes. To further analyse the possible synergistic effect of the combination the set of genes from 1W-ANOVA was subjected to Pearson correlation analysis and hierarchically clustered according to experimental conditions (Fig 5B). The correlation coefficient of LPS vs. LPS+Ibu+Pep was 0.59 and hence lower than the coefficients from LPS vs. LPS+Pep (0.84) and LPS vs. LPS+Ibu (0.84), suggesting stronger dissimilarities between these conditions (see S2 Table for complete list of correlation coefficients).

Hierarchical clustering of the significantly differentially regulated genes resulted in a distinct pattern of gene expression (Fig 5C). Overall, samples stimulated with either Medium or with one of the drugs (i.e. Pep19-2.5 or ibuprofen) clustered together. This suggests that these experimental conditions induce a higher degree of similarity in gene expression pattern compared to the other conditions. Treatment with Pep19-2.5 or ibuprofen alleviated expression levels of most of LPS up-regulated genes (Fig 5C), as indicated by the propensity of these samples to cluster together.

The most remarkable effect on the LPS-induced gene expression pattern was caused by the combined treatment ibuprofen-Pep19-2.5. The gene expression intensity of those genes, which were either induced or repressed by LPS was clearly restored to basal levels after application of ibuprofen and Pep19-2.5 in combination.

### Treatment with Pep19-2.5 and ibuprofen modulates the innate immune response to LPS

**Fig 6:**
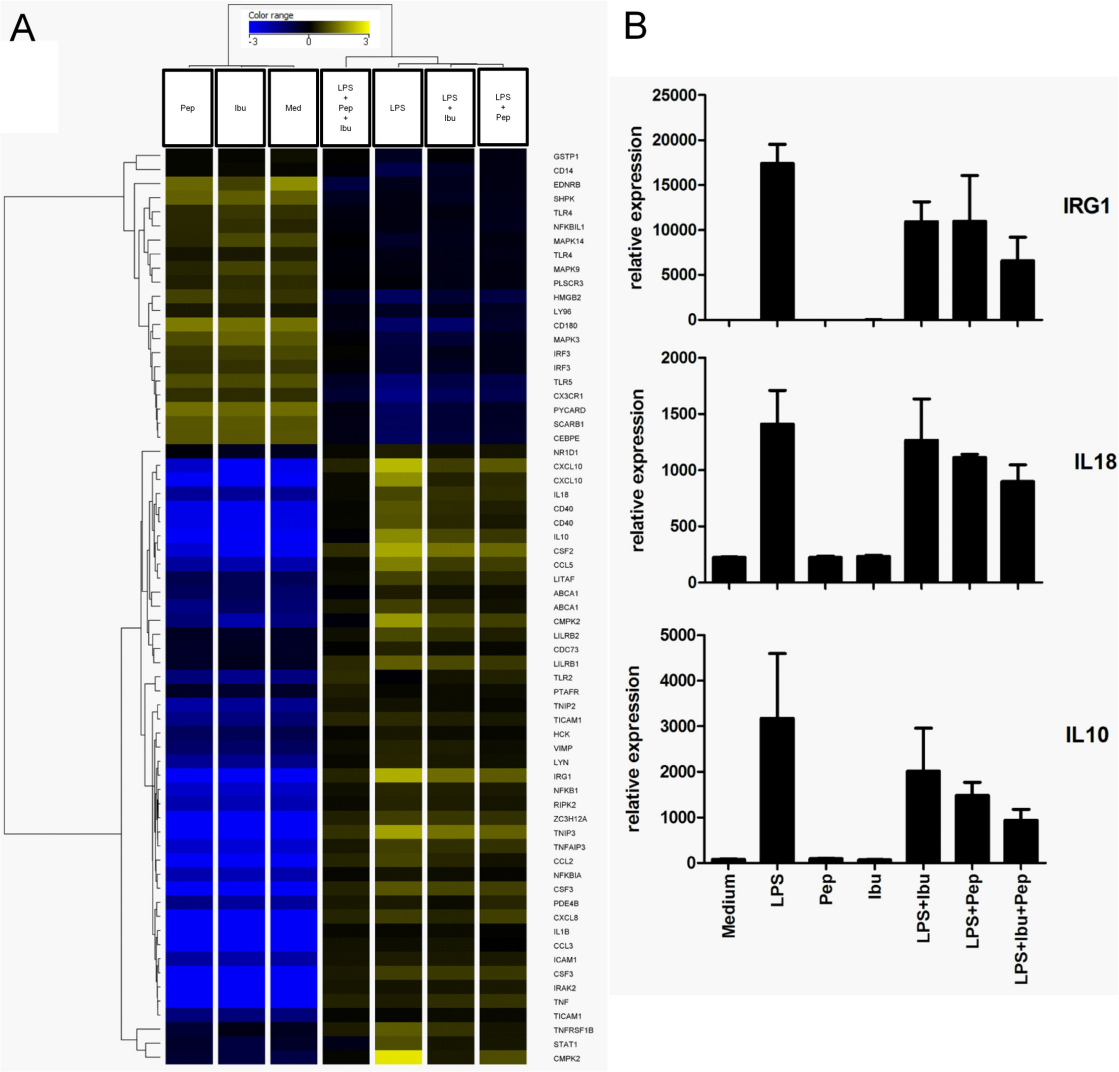
The cellular response to LPS of monocytes is influenced by different combinations of anti-inflammatory drugs. Results from Gene Ontology analysis of data set retrieved from 1W-ANOVA. (A) List of genes belonging to GO term “Cellular Response to LPS” displayed as heatmap of hierarchical clustering (Pearson Centered distance matrix; Ward`s linkage rule) according to their relative expression. (B) Selected single genes from (A) displayed with their relative, quantile-normalized expression.

Gene ontology (GO) analysis was conducted to evaluate the effects of monocytes by LPS stimulation. 23 GO-terms were found to be enriched with a factor of ≥2 (Table 1) and a p-value cut-off ≤10^−7^. Among the enriched GO-terms the most prominent were found to be involved in the innate immune response, pattern recognition receptor signalling and different TLR signalling pathways. Fig 6A displays the genes with the GO term “cellular response to lipopolysaccharide” as a heat-map with hierarchical clustering. A strong response to LPS can be observed of prominent genes such as IRG1, IL18 or IL10 and others indicating a LPS-reactive set of genes. Upon stimulation with both anti-inflammatory drugs, the expression of these genes was more reduced compared to the application of either ibuprofen or Pep19-2.5 (Fig 6B) alone.

**Table 1:** Enriched GO terms among the regulated genes suggest a central involvement of the innate immune response.

GO term	Description	P-value	FDR q-value	Enrichment (N, B, n, b)
GO:0002218	activation of innate immune response	1,98E-015	1,13E-012	2.02 (17671,149,5469,93)
GO:0034142	toll-like receptor 4 signaling pathway	2,23E-015	1,22E-012	2.27 (17671,97,5469,68)
GO:0002758	innate immune response-activating signal transduction	5,50E-015	2,58E-012	2.03 (17671,142,5469,89)
GO:0002221	pattern recognition receptor signaling pathway	2,30E-014	9,17E-012	2.01 (17671,140,5469,87)
GO:0034138	toll-like receptor 3 signaling pathway	2,33E-014	9,02E-012	2.34 (17671,80,5469,58)
GO:0002224	toll-like receptor signaling pathway	2,84E-014	1,07E-011	2.08 (17671,121,5469,78)
GO:0002756	MyD88-independent toll-like receptor signaling pathway	3,11E-013	8,71E-011	2.29 (17671,79,5469,56)
GO:0032480	negative regulation of type I interferon production	4,36E-013	1,12E-010	2.82 (17671,39,5469,34)
GO:0035666	TRIF-dependent toll-like receptor signaling pathway	7,07E-013	1,72E-010	2.30 (17671,76,5469,54)
GO:0034134	toll-like receptor 2 signaling pathway	1,61E-012	3,64E-010	2.30 (17671,73,5469,52)
GO:0032479	regulation of type I interferon production	6,41E-012	1,30E-009	2.03 (17671,108,5469,68)
GO:0038124	toll-like receptor TLR6:TLR2 signaling pathway	8,62E-012	1,64E-009	2.28 (17671,71,5469,50)
GO:0038123	toll-like receptor TLR1:TLR2 signaling pathway	8,62E-012	1,62E-009	2.28 (17671,71,5469,50)
GO:0002755	MyD88-dependent toll-like receptor signaling pathway	1,65E-010	2,55E-008	2.11 (17671,81,5469,53)
GO:0071222	cellular response to lipopolysaccharide	9,79E-010	1,24E-007	2.02 (17671,88,5469,55)
GO:0034166	toll-like receptor 10 signaling pathway	1,15E-009	1,42E-007	2.19 (17671,65,5469,44)
GO:0034146	toll-like receptor 5 signaling pathway	1,15E-009	1,40E-007	2.19 (17671,65,5469,44)
GO:0034162	toll-like receptor 9 signaling pathway	2,00E-009	2,37E-007	2.11 (17671,72,5469,47)
GO:0050688	regulation of defense response to virus	2,73E-009	3,12E-007	2.03 (17671,81,5469,51)
GO:0035872	nucleotide-binding domain, leucine rich repeat containing receptor signaling pathway	3,45E-008	3,29E-006	2.30 (17671,45,5469,32)
GO:0031663	lipopolysaccharide-mediated signaling pathway	2,81E-007	2,17E-005	2.54 (17671,28,5469,22)
GO:0007249	I-kappaB kinase/NF-kappaB signaling	9,05E-007	5,92E-005	2.18 (17671,43,5469,29)
GO:0002753	cytoplasmic pattern recognition receptor signaling pathway	9,51E-007	6,13E-005	2.35 (17671,33,5469,24)

Enrichment (N, B, n, b) is defined as follows: N is the total number of genes. B is the total number of genes associated with a specific GO term. n is the number of genes in the top of the input list or in the target set when appropiate. b is the number of genes in the intersection. Enrichment: (b/n)/(B/N)

### Treatment with Pep19-2.5 and ibuprofen dampens the LPS-induced expression of pro-inflammatory pathways

**Fig 7:**
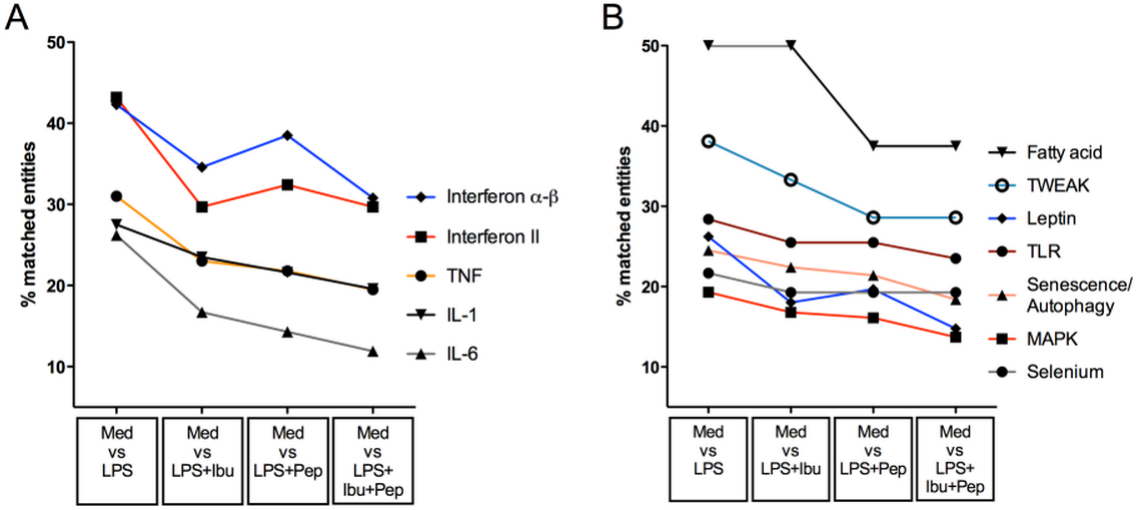
Pep19-2.5 and ibuprofen modulate the LPS-responsive inflammatory signalling pathways. The 10 most significant (lowest p-value) pathways from the LPS stimulation (Med vs LPS) were used as an example for the inflammatory response pattern of monocytes to LPS and compared to the effects of different combinations of anti-inflammatory treatments. Information for pathways is displayed as the % of matched entities to each pathway ([Number of entities found to be significantly enriched in experimental condition/ Total number of entities in pathway]*100). For means of classical LPS-response we included IL-6 signaling_pathway and fatty acid activation which were also found to be significantly enriched upon LPS-stimulation.

We were further interested in the effects of Pep19-2.5 and ibuprofen on underlying signalling pathways in response to LPS. Enrichment analysis was conducted on each experimental condition and the resulting list from LPS stimulation served as standard for comparison of therapeutic efficacy. The 10 most significantly enriched pathways from each condition were compared to the results of single anti-inflammatory treatment or the combination of ibuprofen and Pep19-2.5 (Fig 7). Not unexpectedly, stimulation of monocytes with LPS resulted in a high number of matched entities that belong to pathways involved in the inflammatory response, such as Interferon or TNF and IL-1 (Fig 7A). The overall percentage of matched entities to these pathways was reduced upon administration of ibuprofen or Pep19-2.5. Here, ibuprofen alone exerted its maximal effect on Interferon αβ and Type II Interferon signalling cascades, unlike Pep19-2.5 which exhibited its most potent activity on TNF, IL-1 and IL-6 pathways. In general, the combination of both drugs caused a global down-regulation in genes involved in these pathways. Besides this direct effect at the cytokine expression level, upstream pathways that are involved in general processes were also found to be significantly enriched (Fig 7B). Among these, TLR and TWEAK signalling as well as the MAPK signalling cascade were highly upregulated upon LPS stimulation. In general, treatment with either ibuprofen or Pep19-2.5 dampened to a similar extent the level of matched members with a more significant effect when the compounds were applied in combination. Interestingly, fatty acid activation seemed to be an activity exclusively dependent on the presence of Pep19-2.5. Addition of ibuprofen did not enhance the effect any further.

Overall, combination of both drugs resulted in a reduced percentage of matched genes to these inflammatory pathways which were induced by LPS.

## Discussion

During sepsis treatment the application of NSAIDs is under discussion since decades. Prostaglandin E2 (PGE2) is a lipid-signalling molecule composed of 20-carbons that plays an essential role in homeostasis and inflammatory reactions. The inhibition of prostaglandin synthesis by a blockade of COX enzymes by NSAIDs is commonly used in a wide variety of medical indications. The convenience of administering NSAIDs to septic patients has been under discussion since decades. We investigated in this study the simultaneous anti-septic activity of the NSAID ibuprofen and Pep19-2.5, a promising anti-septic peptide, currently in preclinical development.

Probably, our most important finding is the demonstration that ibuprofen acts in synergy with Pep19-2.5 to stop LPS-dependent mortality *in vivo*. This could be explained by the simultaneous blockage of different cascades involved in inflammatory activation, as investigated here by transcriptome analysis. However, to study the applicability of our findings to sepsis treatment, it will be necessary to test the combinatorial therapy in more realistic animal models induced by the inoculation of viable microorganisms and as supplement to the recommended antibiotic therapy.

Furthermore, we showed that administration of ibuprofen greatly inhibits PEG2 production as expected of a drug down-regulating the COX-dependent pathway. However, this inhibitory effect appears to be insufficient for improving mice survival, and might be due to its suboptimal capacity to reduce TNFα levels *in vivo*, as we showed in Fig 3A. Conversely, Pep19-2.5 appears to have an optimal ability to decrease TNFα levels but it has no inhibitory effect on the production of COX dependent metabolites, such as PGE (Fig 3B). Our results suggest that only when both the COX and the TLR4 dependent pathways are inhibited-as it happens when combining both treatments-, the protection increases significantly. Nevertheless, blocking the TLR4-dependent inflammatory pathway seems to be more relevant for mice survival, in accordance with previous results [11].

Independently from their ability to bind and neutralize microbial targets, some antimicrobial peptides have been reported to have immuno-modulatory activities [27]. Indeed, this would be an attractive property for an agent intended to be used in anti-sepsis therapy. Schuerholz and collaborators showed that, after induction of sepsis in the mouse model of cecal ligation and puncture (CLP), administration of Pep19-2.5 led to marked reduction of CD14 expression in various tissues and to diminished IL-6 as well as IL-1 levels in plasma after 24 h, compared to the untreated control group. Using the same model, these authors showed that this therapeutic activity was even more pronounced than the inflammation inhibiting effect of Polymyxin B, considered as the standard antimicrobial and LPS neutralizing peptide in clinical use [12]. Interestingly this observation comes in line with our findings from the microarray analysis, where Pep19-2.5 tuned down TNF, IL-1 as well as IL-6 pathways of human monocytes in response to LPS.

In this study, the combination of Pep19-2.5 with ibuprofen caused a decrease of TNFα and PGE2 levels in the mouse model of endotoxemia. Particularly important, it led to a drastic advantage in survival compared not only to the untreated mice, but also to the single medications. Since the treatment was administered 1 h after LPS challenge, protection was achieved under therapeutically relevant conditions.

Due to numerous reports in the past concerning the discrepancy of the mouse studies and the situation in humans, especially regarding the use of NSAIDs, we further extended our findings by implementing a comprehensive analysis of *in vitro* LPS-stimulated human cells. Here again a significant reduction in TNFα protein and PGE2 lipid expression levels could be observed by the application of Pep19-2.5 and ibuprofen. The microarray data comes in line with the observations from both, human and mouse experiments. The combined administration of ibuprofen and Pep19-2.5 does not only alleviate the variance as well as expression intensity in global gene expression as indicated in Fig 5, but also restores the expression of those genes which were found to be induced upon LPS stimulation (Fig 5C). A closer examination at the gene ontology term of the cellular response to LPS also indicates the advantage of the combinatorial treatment (Fig 6). Combined administration of Pep19-2.5 and ibuprofen decreases the IL-18 transcription, that was slight in the case of the single Pep19-2.5 treatment, and greatly enhanced upon combination of the peptide with ibuprofen. The IL-18 protein, also known as Interferon-γ inducing factor, has been reported to have pro-inflammatory activity and to play a major role in the development of sepsis [28, 29]. Elevated IL-18 concentrations in the blood are associated with a poor clinical outcome and this biomarker has been proposed as target for therapeutic intervention as well as a potential diagnostic tool.

Interestingly, our data show that combination of Pep19-2.5 and ibuprofen, led to a decrease in LPS induced IL-10 production, an anti-inflammatory cytokine. At present, we do not know whether this is a consequence of the inhibition of the TLR-4 dependent pathway due to LPS neutralization by the peptide or it results from the blockade of PGE2 production by ibuprofen. IL10 reduction caused by the combination is in line with the described role of PGE2 in combination with LPS regarding the induction of an anti-inflammatory phenotype in macrophages characterized by the IL-10 expression [30]. In accordance, the LPS-induced immune responsive gene 1 (IRG1) transcription is reduced by Pep19-2.5 and ibuprofen. The IRG1 protein is expressed during inflammation in macrophages, but its function is not entirely elucidated yet. This protein has been proposed to have antimicrobial [31] and anti-inflammatory activity. A dominance of the latter activity could lead to immunosuppression, which is frequently observed in sepsis patients and constitutes a common risk of death [32]. Since the combined treatment downregulates IRG1, it could have beneficial effects for sepsis therapy.

To further decipher the transcriptomic responses to LPS and the therapeutic effects, Gene Ontology as well as pathway analyses were conducted. The 10 strongest influenced pathways after LPS stimulation compared to medium control were evaluated for the treatment effects (Fig 7). A significant inhibitory effect of the peptide treatment on LPS-induced inflammation was observed for the pathway of fatty acid activation. The most prominent LPS up regulated genes were found to be the very long-chain acyl-CoA synthetase and the long chain fatty acid CoA-ligase 4. The induction of the expression of the acyl-CoA synthetase by LPS in monocytes and macrophages was proposed by Rubinow et al. to play a role in inflammation and innate immunity [33], further fatty acid induced monocyte inflammation can contribute to various chronic inflammatory and cardiovascular diseases [34, 35].

Another interesting pathway, also with regard to its potential use in the classification of the severity of the sepsis disease status is the TWEAK pathway [36]. Here we show that this pathway is also down-regulated in the treated groups and considerably induced during LPS stimulation.

As expected, well established LPS-induced inflammatory pathways and typical markers for the onset of sepsis, such as IL-1, IL-6, TNFα, are also among the pathways with the most significant up regulation after LPS-challenge.

Our work expands previous sepsis-related research with regard to the comprehensive analysis of LPS-induced pathways and demonstrates the protective effect of therapies based on combined anti-inflammatory treatments.

## Supporting Information

S1 Table Significantly differentially expressed genes among the experimental groups. (TXT)

S2 Table Complete table of Pearson Correlation Coefficients for each experimental group. (TXT)
